# Effect of Surfactant and Partial Liquid Ventilation Treatment on Gas Exchange and Lung Mechanics in Immature Lambs**:** Influence of Gestational Age

**DOI:** 10.1371/journal.pone.0056127

**Published:** 2013-02-13

**Authors:** Carmen Rey-Santano, Victoria Mielgo, Elena Gastiasoro, Adolfo Valls-i-Soler, Xabier Murgia

**Affiliations:** 1 Research Unit for Experimental Neonatal Respiratory Physiology, Cruces University Hospital, Barakaldo, Bizkaia, Spain; 2 Neonatal Intensive Care Unit, Cruces University Hospital, Barakaldo, Bizkaia, Spain; University of Giessen Lung Center, Germany

## Abstract

**Objectives:**

Surfactant (SF) and partial liquid ventilation (PLV) improve gas exchange and lung mechanics in neonatal RDS. However, variations in the effects of SF and PLV with degree of lung immaturity have not been thoroughly explored.

**Setting:**

Experimental Neonatal Respiratory Physiology Research Unit, Cruces University Hospital.

**Design:**

Prospective, randomized study using sealed envelopes.

**Subjects:**

36 preterm lambs were exposed (at 125 or 133-days of gestational age) by laparotomy and intubated. Catheters were placed in the jugular vein and carotid artery.

**Interventions:**

All the lambs were assigned to one of three subgroups given: 20 mL/Kg perfluorocarbon and managed with partial liquid ventilation (PLV), surfactant (Curosurf®, 200 mg/kg) or (3) no pulmonary treatment (Controls) for 3 h.

**Measurements and Main Results:**

Cardiovascular parameters, blood gases and pulmonary mechanics were measured. In 125-day gestation lambs, SF treatment partially improved gas exchange and lung mechanics, while PLV produced significant rapid improvements in these parameters. In 133-day lambs, treatments with SF or PLV achieved similarly good responses. Neither surfactant nor PLV significantly affected the cardiovascular parameters.

**Conclusion:**

SF therapy response was more effective in the older gestational age group whereas the effectiveness of PLV therapy was not gestational age dependent.

## Introduction

The introduction of exogenous surfactant (SF) into clinical practice revolutionized the management of neonatal respiratory distress syndrome (RDS), and, more than 30 years later, remains an invaluable tool for the prophylaxis and treatment of neonates with immature lungs.

Generally, intratracheal instillation of exogenous SF to preterm babies produces a rapid improvement in gas exchange, decreases the incidence of pneumothorax and reduces mortality. The effectiveness of SF is, however, affected by clinical variables including exogenous antenatal steroids, lung injury and ventilation, and also by gestational age (GA) of the immature lung. Consequently, a significant percentage of preterm neonates (6–30%) [1], [2] do not respond to SF replacement and for these infants there are currently no other treatment options.

Perfluorocarbon (PFC) liquid ventilation is an experimental therapy with proven efficacy in the treatment of various pulmonary diseases including meconium aspiration, congenital diaphragmatic hernia and neonatal RDS [3]–[5]. PFC liquids are inert organic compounds which have low surface tension, high density and high solubility for oxygen and carbon dioxide but are poor solvents for most other biological compounds, including SF [6]. Intrapulmonary PFCs improve compliance by replacing the gas-liquid interface by a liquid-liquid interface and, due to their high density, gravitate to dependent parts of the lung, reopening collapsed alveoli and redistributing pulmonary blood flow to ventilated regions, thus improving the ventilation-perfusion ratio [7]. In partial liquid ventilation (PLV), a volume of PFC equivalent to the functional residual capacity is instilled into the lung and ventilation is provided with a conventional respirator. Several clinical trials of PLV have been performed in which efficacy results have been more promising in neonatal and pediatric lung injury [8]–[10] than in the adult lung injury [9], [11].

Although several experimental studies have reported gas exchange and lung mechanics results with conventional gas ventilation, SF and PFC therapies at various ages [12], the present study is novel in that it compares these findings in the same RDS model, at two clinically relevant developmental ages, under the control of the same experienced researchers [13]–[15].

While SF and PLV are both intended to reduce surface tension in preterm lungs, PLV might have advantages over SF in very immature lungs, since the larger volumes of PFC used may recruit larger areas and PFCs are not inactivated. We hypothesized that the effects of the PFC on pulmonary gas exchange and mechanics would be similar to or better than those obtained by the surfactant at different GAs. We report the testing of this hypothesis in a single well-defined study under similar controlled conditions, at clinically appropriate ages.

## Materials and Methods

### Ethics Statement

The experimental protocol was performed in the Research Unit of Cruces University Hospital, which is registered in the Official Register of Breeders, Suppliers and Users of animals for experimental and other scientific purposes in the Basque Country, Spain. The protocol complied with all regulations for animal research (EU 86/609 and RD 1201/2005), and was approved by the local Office of Laboratory Animal Welfare of the Cruces University Hospital (Permit Number: EU-03.BI#015_10). All surgery was performed under anesthesia and analgesia, and all efforts were made to minimize suffering.

### Animal Preparation and Preterm Delivery

Date-mated Latxa ewes (term gestation: 153±2 days) were prepared for caesarean section by injecting xylazine (0.15 mg/kg, intramuscularly), ketamine (5 mg/kg, intravenously), and anesthesia maintained with propofol (30–40 mg/kg/h, intravenously), with Ringeŕs lactate infused as needed [13]. A tube was inserted into the trachea and connected to a volume-controlled ventilator, to maintain adequate gas exchange. Initial settings were: respiratory frequency (f_R_) 30 cycles/min; Peak Inspiratory Pressure/Positive End-Expiratory Pressure (PIP/PEEP) 14–16/3 cmH_2_O; inspiratory (I): expiratory (E) ratio 1∶2; and inspiratory fraction of oxygen (F_iO2_) 0.4–0.5. A cannula was inserted into a peripheral arterial to monitor mean arterial pressure (MABP), heart rate (HR) and arterial blood gases [13].

In total, 18 lamb fetuses were delivered at a GA of 125 days (81% of term) and another 18 at 133 days (86% of term). With the ewe lying on its right side, the uterus was exposed by a lateral subcostal incision. We inserted 3.0- and 4-mm tracheal tubes (Hi-Lo Jet Tracheal tube, Mallinckrodt Medical, St. Louis, MO) by tracheotomy for lower and higher GA lambs respectively, and these were tied around the trachea to prevent leaks [13]. End-hole catheters (5 Fr) were inserted into the jugular vein and carotid artery (Umbilical catheter, Vygon, Ecouen, France). Lambs were given intravenous ketamine (3 mg/kg) and pancuronium bromide (0.1 mg/kg), and the umbilical cord was cut [13].

### Postdelivery Management

Immediately after delivery, lambs were weighed, dried and placed under radiant warmers to maintain the rectal temperature at 38–39°C. Then, the tracheal tube was connected to a neonatal time-cycled, pressure-limited ventilator (BP-200, Beard Medical Systems, Riverside, CA), with the following initial settings: f_R_ 60 cycles/min; PIP/PEEP 30/5 or 35/5 cmH_2_O, for the 133- and 125-days GA lambs, respectively; I:E ratio 1∶2; F_iO2_ 1.0, and flow rate 10 l/min. The value of F_iO2_ was kept constant throughout the experiment, the other parameters being changed to maintain Pa_CO2_ values at 35–45 mmHg with a maximal PIP of 40 cmH_2_O to avoid pneumothorax. Ketamine (5 mg/kg/h) and fentanyl (4 ug/kg/min) were infused in 5% glucose to maintain anesthesia and analgesia, and atracurium besylate (1.2 mg/kg) to prevent spontaneous breathing. If needed, dopamine was infused at 5–10 ug/kg/min to maintain a MABP of 40 mmHg. Bicarbonate solution was administered intravenously (intermittently in ≤2 meq/kg boluses) if the pH fell to <7.25 to manage metabolic acidosis and non-bicarbonate buffering [tris(hydroxymethyl)aminomethane (THAM 0.5 M) was used to correct respiratory acidosis. The amount of supplemental bicarbonate or THAM required was calculated as follows:
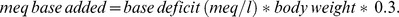



### Experimental Design

The ewes, having been date-mated for delivery at two gestational ages (125 or 133 days) were randomly allocated to two delivery groups (one at each gestational age) and within these to one of the three treatment subgroups using sealed envelopes. All lambs were first stabilized on the ventilator (30 min), and then given one of the following treatments:

- Surfactant (SF) groups, SF-125 d and SF-133 d (n = 6 in each): lambs received 200 mg/kg of Curosurf® (Chiesi Farmaceutici, Parma, Italy; 80 mg/mL) in supine position instilled as per its label, without being disconnecting from the ventilator, and were ventilated for 3 h.
- Partial liquid ventilation (PLV) groups, PLV-125 d and PLV-133 d (n = 6 in each): lambs received an intra-tracheal dose of 20 ml/kg Perfluorodecalin (PFD, F2 Chemicals, Lancashire, UK); density at 25°C = 1.95 g/ml; vapor pressure at 37°C = 14 mmHg; surface tension = 15 dyne/cm and oxygen solubility = 49 mL/100 ml), and ventilated for 3 h. For each lamb, one third of the total dose was instilled as a bolus with the animal in the supine, left-side and right-side positions to enhance PFC distribution. Ventilator frequency and PIP were briefly adjusted to obtain a good PFC movement across the tracheal tube until PFC distributed into the lungs and the meniscus stabilized. PEEP was maintained constant at 5 cmH_2_O along the experiment. However, at settled time intervals PEEP was briefly withdrawn (PEEP = 0 cmH_2_O) to determinate whether a PFC meniscus was present or not at the tracheal tube at end expiration in order to ensure a proper PFC load equivalent to the functional residual capacity. To compensate for evaporation losses of PFC, the initial amount was maintained by continuous infusion at a rate of 2.5 ml/kg/h via a side tube on the endotracheal tube. During partial liquid ventilation, no suction was applied to the endotracheal tube.
- Control groups, Control-125 d and Control-133 d (n = 6 in each): lambs did not receive SF or PFC but were ventilated for 3 h.

### Measurements

HR and MABP were continuously measured and recorded (OmniCare, GMS 24, Hewlett Packard, Böblingen, Germany). The Oxygenation Index (OI) was calculated as follow: OI = [mean airway pressure (MAP) (cmH_2_O) * F_IO2_/Pa_O2_(mmHg)*100] and Ventilation Efficiency Index (VEI) as: VEI = 3800/(PIP-PEEP) *ventilator rate *Pa_CO2_ [16]. Arterial pH, Pa_CO2_, base excess, pH (AVL 945, AVL Biomedical Instruments, Schaffhausen, Switzerland), OI and VEI were measured before pulmonary treatment (Baseline), and at 15 and 30 min, and then every 30 min until the end of experiment (3 h).

Lung dynamic compliance (C_dyn_) and tidal volume (V_T_) were calculated by a computerized system (Peds®, MAS Lab, PTI Ltd, Hatfield, PA) [15], [17]. Airflow was measured during the whole respiratory cycle with a pneumotachometer (Fleish 00, OEM Medical, Richmond, VA), and pressure with a differential pressure transducer (MP45, Validyne Engineering, Northridge, CA). Data for each breath were automatically screened to confirm that they met all criteria for selection of the breath before being included in the averaged data. In order to avoid the effect of perfluorocarbon vapor on the measurement of airflow and tidal volume, the lung function measurements were based only on the inspiratory flow [18]. Ten random breaths were analyzed to represent pulmonary function for each lamb at each time point interval. Lung mechanics were measured before pulmonary treatment (Baseline), and every 30 min until the end of experiment (3 h).

### Statistical Methods

Data are reported as mean±SEM. Results were assessed using Levenés tests, to confirm the homogeneity of variance between the treatments, and Kolmogorov-Smirnoff tests for normality (JMP 8, Statistical Discovery, SAS, NC). One-way ANOVA was performed to assess time point differences in gas exchange, systemic hemodynamic parameters and lung mechanics as a function of group. Comparisons of results at all time points were performed by two-way repeated-measures ANOVA as a function of group and time. Post hoc multiple comparisons were performed with the Bonferonni Dunn test where appropriate. A *P<*0.05 was accepted as significant.

## Results

Table 1 summarises the fetal (in utero) and baseline (pre-treatment) characteristics of the lambs. There were no significant differences between control and pulmonary treated groups at either of the GAs studied in body weight, fetal blood gas and hemodynamic values or baseline arterial blood gas, hemodynamic and lung mechanics values. On the other hand comparing lambs at the two GAs, there were significant differences in body weight, baseline arterial blood gas and hemodynamic values (Table 1). The 125-day-GA lambs had poorer baseline blood gas values and OI, even when higher mean airway pressure was applied, compared to those delivered at 133 days.

**Table 1:** Characteristics of preterm lambs during fetal life and before pulmonary treatment at the two gestational ages studied.

	Delivered at 125±1 days GA	Delivered at 133±1 days GA	P value
Number of animals	18	18	NS
Weight (kg)	2.7±0.3	3.5±0.2	0.03
Fetal values (in utero)			
Arterial blood gases			
pH	7.30±0.01	7.30±0.04	NS
Pa_O2_ (mmHg)	23±2	27±2	NS
Pa_CO2_ (mmHg)	46±9	48±3	NS
Hemodynamic values			
MABP (mmHg)	59±3	56±2	NS
HR (beats/min)	177±7	152±5	0.008
Baseline values			
Arterial blood values			
pH	6.95±0.05	7.1±0.02	0.045
Pa_O2_ (mmHg)	17±4	38±8	0.027
Pa_CO2_ (mmHg)	94±5	80±5	NS
OI	151±13	86±18	0.012
VEI	0.033±0.003	0.036±0.004	NS
Hemodynamic values			
MABP (mmHg)	62±3	72±3	0.018
HR (beats/min)	155±12	172±11	NS
Lung mechanics			
C_dyn_ (ml/cmH_2_O/kg)	0.0757±0.0055	0.1120±0.0179	NS
V_T_ (ml/kg)	2.04±0.22	2.43±0.35	NS
MAP	15±0.2	13±0.4	0.002

Data are presented as mean±SEM. C_dyn_: dynamic compliance; GA: gestational age; HR: heart rate; MABP: mean arterial blood pressure; MAP: mean airway pressure; OI: oxygenation index; PLV: partial liquid ventilation; SF: surfactant; VEI: ventilation efficiency index; V_T_: tidal volume; NS: non-significant. *P* value indicates significant differences in body weight, in baseline pre-treatment blood gas (Baseline), in hemodynamic and in mean airway pressure values between animals at the two GAs (one-way ANOVA).

Survival rates were similar; one lamb in each GA group did not reach the end of the experiment, dying at 60 and 120 min in the SF-125 d and Control-135 d groups, respectively. These two lambs died following cardiac arrest, after a period of extremely low MABP that was unresponsive to infusions of both volume expanders and dopamine (up to 10 ug/kg/min).

### Pulmonary Gas Exchange

**Figure 1:**
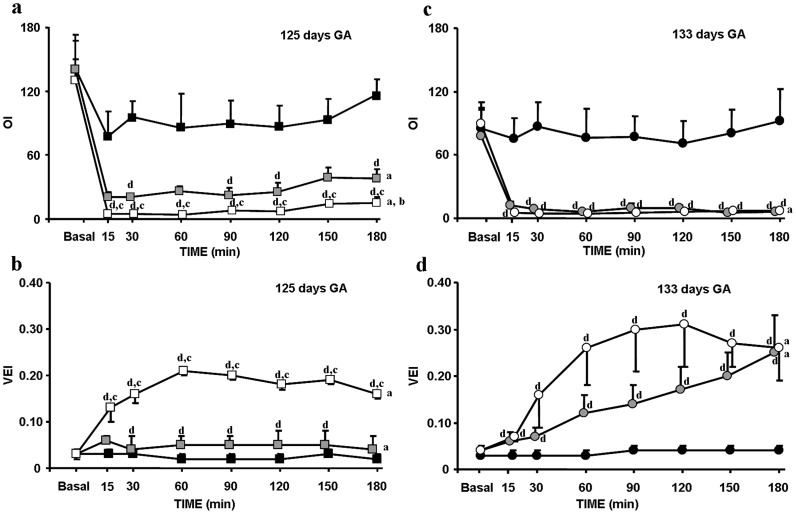
Mean OI and VEI in premature lambs at two gestational ages. Mean OI (a) and mean VEI (b) at 125 days of GA in Control-125d [indicated by black squares]; surfactant, SF-125d [indicated by grey squares] and partial liquid ventilation, PLV-125d [indicated by white squares] groups. Mean OI (c) and mean VEI (d) at 133 days of GA in Control-133d [indicated by black circles]; surfactant, SF-133d [indicated by grey circles]; and partial liquid ventilation, PLV-133d [indicated by white circles] groups. Fetal: indicates the fetal values in both groups, while the lambs are still connected to the placenta. Baseline: shows pre-treatment values before pulmonary treatment. Values are given as mean±SEM. Significant differences are indicated by: (^a^) vs. Control group, *P*<0.05, two-way ANOVA over all time points; (^b^) vs. SF group, *P*<0.05, two-way ANOVA over all time point; (^c^) vs. SF group, *P*<0.05, one-way ANOVA at each time point; and (^d^) vs. Control group, *P*<0.05, one-way ANOVA at each time point.

**Figure 2:**
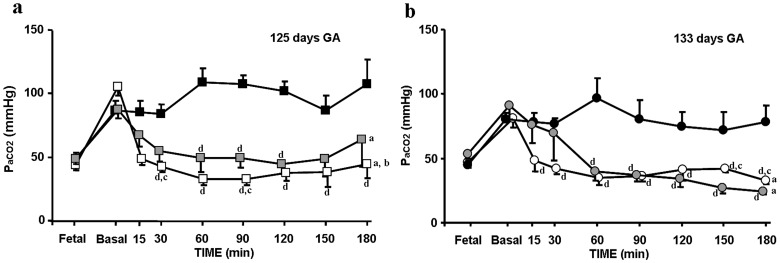
Mean arterial Pa_CO2_ in premature lambs at two gestational ages. Mean arterial Pa_CO2_ (a) at 125 days of GA in Control-125d [indicated by black squares]; surfactant, SF-125d [indicated by grey squares]; and partial liquid ventilation, PLV-125d [indicated by white squares] groups. Mean Pa_CO2_ (b) at 133 days of GA in Control-133d [indicated by black circles]; surfactant, SF-133d [indicated by grey circles]; and partial liquid ventilation, PLV-133d [indicated by white circles] groups. Fetal: indicates the fetal values in both groups, while the lambs are still connected to the placenta. Baseline: shows pre-treatment values before pulmonary treatment. Values are given as mean±SEM. Significant differences are indicated by: (^a^) vs. Control group, *P*<0.05, two-way ANOVA over all time points; (^b^) vs. SF group, *P*<0.05, two-way ANOVA over all time point; (^c^) vs. SF group, *P*<0.05, one-way ANOVA at each time point; and (^d^) vs. Control group, *P*<0.05, one-way ANOVA at each time point.

The premature lambs delivered at 125 days of gestation developed very severe RDS manifested by hypercarbia (>90 mmHg), hypoxemia (<20 mmHg) and very high OI (>100) despite mechanical ventilation with high inspired oxygen concentrations (F_IO2_∶1) (Table 1). After treatment, there were significant decreases in mean OI and in Pa_CO2_ and increases in VEI in SF-125d compared to the Controls at 30–60 min after SF instillation (Figures 1A, 2A and 1B, respectively). However, OI values remained above 30, VEI below 0.10 and Pa_CO2_ above 50 mmHg throughout the experiment. In PLV-125d, there were a significant decreases in OI and in Pa_CO2_ and increases in VEI at 15–30 min after tracheal perfluorocarbon instillation compared to Control and SF-125d groups; and OI values remained below 15, VEI above 0.20 and Pa_CO2_ in the normal range throughout (Figures 1A, 2A and 1B, respectively).

Mean arterial pH decreased after delivery from 7.3±0.01 to 6.9±0.05 before pulmonary rescue treatment. Following SF and PFC instillation, the pH increased, so that by 15 min values were significantly higher in both groups (SF-125d: 7.18±0.03; PLV-125d: 7.31±0.05 vs. Control-125d: 6.95±0.05) and remained constant at around 7.35 throughout the experiment.

The premature lambs delivered at 133 days of gestation also developed RDS manifested by hypercarbia (>70 mmHg), hypoxemia (<50 mmHg) and high OI (>60) with F_IO2_∶1 though (as the data show) their condition was less severe than that of those delivered at the earlier GA (Table 1). After pulmonary treatment, SF-133d and PLV-133d groups showed significant decreases in mean OI and Pa_CO2_ and increases in VEI compared to the Control group at 15–30 min (Figures 1C, 2B, 1D). In both groups, OI values remained below 10, VEI above 0.15 and Pa_CO2_ in the normal range throughout, OI values being lower and VEI values higher in the PLV-133d group (Figures 1C, 1D).

In these lambs, mean arterial pH also decreased after delivery from 7.3±0.01 to 7.1±0.02 before treatment. Following SF and PFC instillation, the pH increased in both groups, so that by 15 min values were significantly higher than baseline in both groups, and remained constant at around 7.4 to the end of the experiment.

### Cardiovascular Profile

After delivery and pulmonary rescue therapy, MABP was statistically similar in all groups at all time points (Table 2), though it tended to decrease after 60 min of gas ventilation in Control-125d lambs. Although, the PLV-125d group had lower HR than Control-125d and SF-125d groups for the first minutes of treatment; after that, values remained stable and similar to those in the other groups until the end of the experiment (Table 2).

**Table 2:** Hemodynamic parameters in preterm lambs at two gestational ages.

	Delivered at 125±1 days GA			Delivered at 133±1 days GA		
	Contr-125d	SF-125d	PLV -125d	Contr-133d	SF-133d	PLV-133d
Weight (kg)	2.7±0.5	2.4±0.3	3.0±0.3	3.5±0.3	3.4±0.1	3.5±0.1
MABP (mmHg)						
Baseline	69±12	62±7	61±4	60±5	70±4	76±6
Post-treatment						
15 min	69±12	58±3	66±5	60±5	69±3	58±3^c^
30 min	69±7	64±4	67±8	65±5	72±2	62±4^c^
60 min	71±6	56±6	63±5	60±4	63±3	58±3
90 min	65±8	57±6	65±6	53±4	64±4	63±6
120 min	60±8	53±4	62±4	50±5	70±4	57±4
150 min	52±6	56±7	59±5	57±4	59±4	54±4
180 min	44±5	57±3	60±7	54±4	61±5	54±4
HR (beats/min)						
Baseline	145±23	174±27	147±13	144±19	188±13	188±13
Post-treatment						
15 min	211±6	169±9^d^	145±8^d^	181±8	179±11	142±9^d, c^
30 min	211±8	171±9^d^	152±11	185±11	166±14	136±9^d^
60 min	207±10	192±24	194±17	176±8	164±12	145±5^d^
90 min	200±20	207±16	220±21	169±12	179±16	168±10
120 min	198±15	197±14	218±21	175±21	205±20	175±19
150 min	208±15	219±13	197±17	186±20	179±13	180±16
180 min	202±20	213±11	209±25^a, b^	178±24	187±12	175±11^a^

Data are presented as mean±SEM. GA: gestational age; HR: heart rate; MABP: mean arterial blood pressure; PLV: partial liquid ventilation; SF: surfactantSignificant differences are indicated by:

(a) vs. Control group, *P*<0.05, two-way ANOVA over all time points;

(b) vs. SF group, *P*<0.05, two-way ANOVA over all time point;

(c) vs. SF group, *P*<0.05, one-way ANOVA at each time point; and

(d) vs. Control group, *P*<0.05, one-way ANOVA at each time point. One-way ANOVA test was used to compare the body weight parameter between animals at different groups at the two GAs.

In the 133-day GA lambs, HR transiently decreased following PFC instillation for the first few minutes compared to the Control group, while MABP remained stable (Table 2). Surfactant treatment did not significantly affect the cardiovascular parameters (Table 2).

### Pulmonary Mechanics

**Figure 3:**
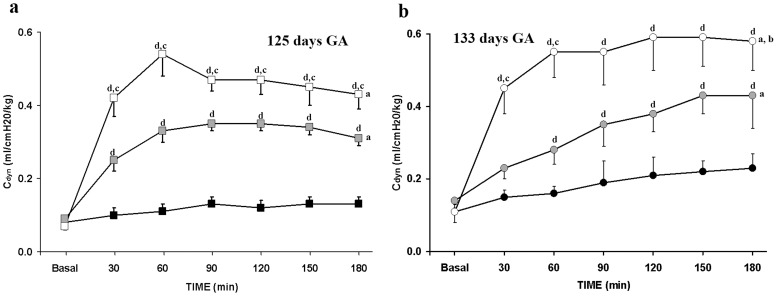
Pulmonary mechanics in premature lambs at two of gestational ages. Mean dynamic compliance; C_dyn_ (a) at 125 days of GA in Control-125d [indicated by black squares]; surfactant, SF-125d [indicated by grey squares]; and partial liquid ventilation, PLV-125d [indicated by white squares] groups. Mean C_dyn_ (b) at 133 days of GA in Control-133d [indicated by black circles]; surfactant, SF-133d [indicated by grey circles]; and partial liquid ventilation, PLV-133d [indicated by white circles] groups. Fetal: indicates the fetal values in each group, while the lambs are still connected to the placenta. Baseline: shows pre-treatment values before pulmonary treatment. Values are given as mean±SEM. Significant differences are indicated by: (^a^) vs. Control group, *P*<0.05, two-way ANOVA over all time points; (^b^) vs. SF group, *P*<0.05, two-way ANOVA over all time point; (^c^) vs. SF group, *P*<0.05, one-way ANOVA at each time point; and (^d^) vs. Control group, *P*<0.05, one-way ANOVA at each time point.

In the 125-day GA lambs, the initial mean C_dyn_ was extremely low in all groups (0.0757±0.0055 ml/cmH_2_O/kg) (Table 1). A significant upwards trend was observed in the SF-125d group for the first 90 min of the experiment, values (0.35 ml/cmH_2_O/kg) remaining higher than controls of this GA until 3 h (Figure 3A). In PLV-125d, there was a significant increase in C_dyn_, recorded at 30 min after tracheal PFC instillation compared to Control and SF-125d groups; and the latter maintained values over 0.45 ml/cmH_2_O/kg throughout the experiment (Figure 3A). V_T_ followed a similar pattern to that of C_dyn_: it was initially low in all groups (2.04±0.22 ml/kg) (Table 1), significantly increased after surfactant instillation during the first 90 min of experiment and remained at these levels (8.0±0.5 ml/kg) until 3 h. In the PLV-125d group, V_T_ increased up to 90 min, and stayed over 10 ml/kg until the end of experiment.

In the 133-day GA lambs, initial mean C_dyn_ was very low in all groups (0.1120±0.0179 ml/cmH_2_O/kg) (Table 1). It then increased significantly over the experiment in the SF-133d group (0.43 ml/cmH_2_O/kg at 3 h) (Figure 3B). In the PLV-133d group, there was also a significant increase in C_dyn_, at 30 min after tracheal PFC instillation compared to Control and SF-133d groups; and it continued increasing until 120 min, maintaining values (0.6 ml/cmH_2_O/kg) until 3 h (Figure 3B). V_T_ followed a similar pattern to that of C_dyn_: it was initially low in all groups (2.43±0.35 ml/kg), continuously increasing after surfactant instillation until 3 h. In the PLV-133d group, V_T_ increased up to 60 min and remained over 10 ml/kg until the end of experiment.

## Discussion

Our study shows that immature lambs with severe respiratory failure at different gestational ages do have different responses to surfactant replacement therapy or partial liquid ventilation. While PLV therapy improves gas exchange and pulmonary function independently of GA, response to SF rescue therapy differed between the two GAs studied, more immature animals having poor response.

In this study with the Basque Latxa breed, lungs were more immature at 125 and 133 days of GA than observed previously in other sheep breeds used as model of RDS [13]. Extrapolating the C_dyn_ values at baseline in the Latxa lambs to the correlations between C_dyn_ and GA described by Born et al. [19] for Suffolk sheep and Wolfson et al. [20] for Western sheep, the estimated GA for the lambs used in the present study would be 118±3 and 95±2 days for the lambs delivered at 133 and 125 days respectively. According to this correction, the lung development of the lambs delivered at 133 days would be between the late canalicular and saccular stages, while those delivered at 125 days would be the early-middle canalicular stage [21].

A similar pattern of lung development is observed across mammalian species, although the timing and onset of each stage vary considerably based on the length of the gestational period. The phases of fetal lamb lung development are as follows: embryonic, 0–40 days (0–7 week of gestation in humans); pseudoglandular, 40–80 days (8–16 weeks); canalicular, 80–120 days (17–27 weeks); saccular, 120 days to term at 150 days of gestation (28 weeks to term at 42 weeks of gestation in humans) [21]–[23].

In RDS, the degree of prematurity is of great importance as it may determine the success of pulmonary rescue treatment, lung immaturity being related to a younger gestational age. In our study, a reduction of 8–9 days in the GA resulted in a significantly lower pH, arterial Pa_O2_ and MABP at baseline (125-day GA lambs). Moreover, the arterial Pa_CO2_, OI and C_dyn_ values at this earlier time point were markedly poorer despite the higher mean airway pressure used in this group, reflecting the lung being less mature in the 125-day than the 133-day GA lambs.

These differences might account for the different responses to SF instillation in the two groups. After SF treatment of lambs delivered at 125-days of GA, gas exchange and lung mechanics improved significantly compared to the untreated controls, but these positive effects were limited (Pa_O2_<100 mmHg; OI>30; Pa_CO2_>50 mmHg with F_IO2_∶1.0). Similar poor responses to SF have been described previously [24]–[26], partial or transient gas exchange improvement being observed with little change in lung mechanics. We speculate that the limited response to SF in lambs delivered at 125 days of GA is influenced by an immature lung anatomical structure [21], poor initial SF distribution due to low lung volume, rapid inactivation of SF starting as soon as a few minutes after the onset of mechanical ventilation [27], limited biochemical maturity of the type II pneumocytes showing weaker metabolic capacities and inadequate control of oxidative stress and inflammation [28]. Such factors could contribute to a reduction in or lack of response to SF treatment [1], [2] and the need for repeat doses of SF in infants with RDS [29]. In our study, the administration of further doses of SF could have improved the effectiveness of treatment in the SF-125d group [30].

The good response to SF rescue therapy in the 133-day GA group produced a rapid increase in arterial oxygenation and lung mechanics [31]. With the slight increase in GA, of just 8 days, the therapeutic response to SF was adequate, probably due to improvements in the initial SF distribution and maturation of metabolic pathways [28] and anatomical structures of the lung [21].

The pulmonary response to PLV resulted in a significant improvement in oxygenation and lung mechanics in lambs delivered at 125 days of GA compared to both untreated and SF-treated animals. The difference in response between SF-125d and PLV-125d could be explained by a difference in birth weight (related to immaturity). However, our ewes were date-mated (equal GA) and birth weights in the groups were not significantly different (2.4±0.3 vs. 3.0±0.3 kg, ns). Moreover, the improvement in gas exchange and lung function after intratracheal PFC was of the same magnitude in all lambs regardless of the GA of the PLV-treated lambs. Therefore, in our study the mechanism by which intratracheal instillation of PFC achieved a good response was independent of GA, unlike the case of SF replacement.

In PLV, a greater initial filling of the lungs with a high-density compound allows a rapid recruitment of lung tissue, since PFCs gravitate to the dependent zones [32], [33]. In addition, intrapulmonary PFC keeps the alveoli mechanically open acting as liquid PEEP and redistributing the pulmonary blood flow to the ventilated zones, thus improving ventilation-perfusion mismatch [34]. Further, replacement of the alveolar-membrane gas-liquid interface with a liquid-liquid interface improves lung compliance by minimizing the surface tension forces related to SF deficiency. Together with these mechanisms, the high solubility for oxygen and carbon dioxide ensures a proper gas-exchange and the inert nature of PFCs means that they are not affected by inactivation and mobilizes alveolar and bronchiolar exudates and debris to the trachea where they can be removed by suction [8]. The cumulative effects of these mechanisms might be responsible for the effectiveness of PLV treatment independent of the GA.

In lambs delivered at 133 days a good response was achieved with both SF and PLV treatments without meaningful changes in systemic hemodynamics [35]–[38]. In terms of oxygen levels, oxygenation was significantly better than in controls with both treatments, without significant differences between SF and PLV groups. The reduction of Pa_CO2_ to normal levels was, however, faster with PLV, through a rapid and significantly greater improvement in lung mechanics. This is a key factor in preterm neonates since the arterial Pa_CO2_ is a potent regulator of cerebral vascular tone [39]. On the other hand, there was a tendency for a brief delay in the effect of SF on C_dyn_. This might be explained by the transient increase in expiratory resistance over the first 30 minutes following SF instillation (data not shown) which might have delayed the distal migration of SF resulting in partial transient SF deposition in conducting airways and, therefore, reduced alveolar recruitment.

The use of SF replacement therapy and prenatal steroids has substantially improved the clinical course of some preterm infants, but not all respond [1], [2], response being especially poor among the most immature infants. Experimental PFC liquid ventilation emerged as a promising new technique to address ventilation problems in humans [7]. The encouraging data from our study suggest that this technique could indeed be very useful in neonatal respiratory distress associated with severe prematurity. Several clinical trials of PLV have demonstrated its efficacy in neonatal and pediatric lung injury [8]–[10]. However, this technique has been less promising in adult lung injury [9], [11], and, while there was one clinical trial of the PLV technique, there have been no further trial [40]. Currently, research is ongoing into the most effective way of providing PFC-assisted ventilation. The use of a single small dose (<5 ml/kg) to facilitate initial lung recruitment or aerosolized PFC may result in better patient outcomes with lower adverse effects [41], [42].

Despite our findings showing a better response to PLV compared to SF in very preterm lambs, some questions remain unanswered. *Limitations* of this *study* include, firstly, the fact that the histology of the lung and distribution of PFC within the lungs were not assessed in the present work. Specifically, PFCs are immiscible in water and to our knowledge no substances can be dissolved in them; therefore, the methods previously used by our group to study pulmonary distribution were not applicable. In addition, the experimental design was focused on the acute efficacy of SF therapy and PLV and, for that reason, the observational period was only up to 180 minutes. Further studies should focus on PFC replacement [43], interaction between PLV and SF [44], and improving the transition from liquid to gas respiratory support.

In summary, our data show that a reduction in GA produced significantly more severe RDS in our preterm lamb model, and that treatment with PLV was able to improve lung function to the same extent independent of the GA. In contrast, while the SF treatment was effective in animals delivered at 133 days of GA (although slightly less so than PLV), SF instillation produced a poor response in animals at a younger GA, with less developed lungs. Overall, we conclude that it is worth continuing to explore the hypothetical use of PFCs in a clinical setting as it might help in the treatment of babies with poor lung development who do not respond to SF; that is, PFCs could be considered as a rescue therapy when the conventional therapies have failed.
